# Esophageal Cancer: Genomic and Molecular Characterization, Stem Cell Compartment and Clonal Evolution

**DOI:** 10.3390/medicines4030067

**Published:** 2017-09-14

**Authors:** Ugo Testa, Germana Castelli, Elvira Pelosi

**Affiliations:** Department of Hematology, Oncology and Molecular Medicine, Istituto Superiore di Sanità, 00141 Rome, Italy; germana.castelli@iss.it (G.C.); elvira.pelosi@iss.it (E.P.)

**Keywords:** esophageal cancer, Barrett’s disease, cancer stem cells, tumor xenotrasplantation assay, gene sequencing, gene expression profiling

## Abstract

Esophageal cancer (EC) is the eighth most common cancer and is the sixth leading cause of death worldwide. The incidence of histologic subtypes of EC, esophageal adenocarcinoma (EAC) and esophageal squamous carcinoma (ESCC), display considerable geographic variation. EAC arises from metaplastic Barrett’s esophagus (BE) in the context of chronic inflammation secondary to exposure to acid and bile. The main risk factors for developing ESCC are cigarette smoking and alcohol consumption. The main somatic genetic abnormalities showed a different genetic landscape in EAC compared to ESCC. EAC is a heterogeneous cancer dominated by copy number alterations, a high mutational burden, co-amplification of receptor tyrosine kinase, frequent TP53 mutations. The cellular origins of BE and EAC are still not understood: animal models supported a cellular origin either from stem cells located in the basal layer of esophageal epithelium or from progenitors present in the cardia region. Many studies support the existence of cancer stem cells (CSCs) able to initiate and maintain EAC or ESCC. The exact identification of these CSCs, as well as their role in the pathogenesis of EAC and ESCC remain still to be demonstrated. The reviewed studies suggest that current molecular and cellular characterization of EAC and ESCC should serve as background for development of new treatment strategies.

## 1. Introduction

Esophageal cancer represents the sixth most common cause of cancer death and the eighth in incidence worldwide. In fact, it accounts for about 4% of cancer diagnoses and for 6% of cancer deaths. The prognosis for esophageal carcinoma is poor, with a 5-year survival rate of 19% and only 0.9% for advanced esophageal carcinoma. The incidence and histologic subtypes of esophageal cancer display a considerable geographic variation. Esophageal squamous cell carcinoma (ESCC) is the most frequent esophageal cancer in the world, with the highest incidence in eastern Asia and parts of Africa. The major risk factors for ESCC are represented by tobacco and alcohol consumption, but also other environmental factors play a role in the development of this cancer, such as the consumption of hot beverages, nutritional deficiencies and limited intake of fruits and vegetables [1,2,3]. In contrast, esophageal adenocarcinoma (EAC) is the most frequent subtype in Western countries and is one of the cancers whose frequency continues to increase [1,2,3]. The reasons for its increase seem to be mainly related to the increasing incidence of gastro-esophageal reflux disease (GERD) and obesity [1,2,3]. It was estimated that 20% of adult Americans have chronic GERD.

Most patients with an esophageal cancer have a disease at an advanced stage and, therefore, are not eligible for curative therapy or develop tumor recurrence, in spite treated with a curative intent. The treatment of esophageal cancer is mainly based on surgery. Radiotherapy, chemotherapy or chemoradiotherapy in both EAC and ESCC are of limited efficacy; therefore, there is an absolute need for an improvement of medical treatment through the identification of new therapeutic targets [2,3].

Developments in high-throughput genomic technologies have led to a better understanding of the disease heterogeneity and of the molecular basis underlying the development of EAC and ESCC [4]. These studies have indicated that ESCC is more similar to other squamous cancers, such as those of head and neck, than to EAC; on the other hand, EAC is more similar to the chromosomally unstable subtype of gastric carcinoma than to ESCC [2,3,4]. The importance of studies of genomic characterization of these tumors is not limited only to a better understanding of disease pathogenesis, but also represents a unique and precious tool for the identification of new therapeutic targets.

In spite of the efforts to molecularly characterize esophageal cancer have led to the identification of patient subgroups who may potentially benefit from target therapies, the therapeutic success so far obtained was very limited. Thus, only the targeting of HER2-positive tumors was associated with some clinical efficacy [5,6], while other therapies targeting the epidermal growth factor receptor or the mesenchymal-epithelial transition pathways have been largely unsuccessful.

The present review will analyze the recent developments in our understanding of the molecular (somatic genetic alterations) and cellular (tumor initiating cells) basis underlying EAC and ESCC. Only an integrated genomic and cellular characterization at the level of single esophageal cancer, considering the major driver mutations, the tumor heterogeneity and the major biochemical pathways sustaining tumor survival and proliferation, may led to the identification of clinically suitable biomarkers and to drive the development of new multitargeting therapeutic approaches.

## 2. Genetic Alterations of Esophageal Cancer

### 2.1. Molecular Abnormalities of EAC

**Figure 1:**
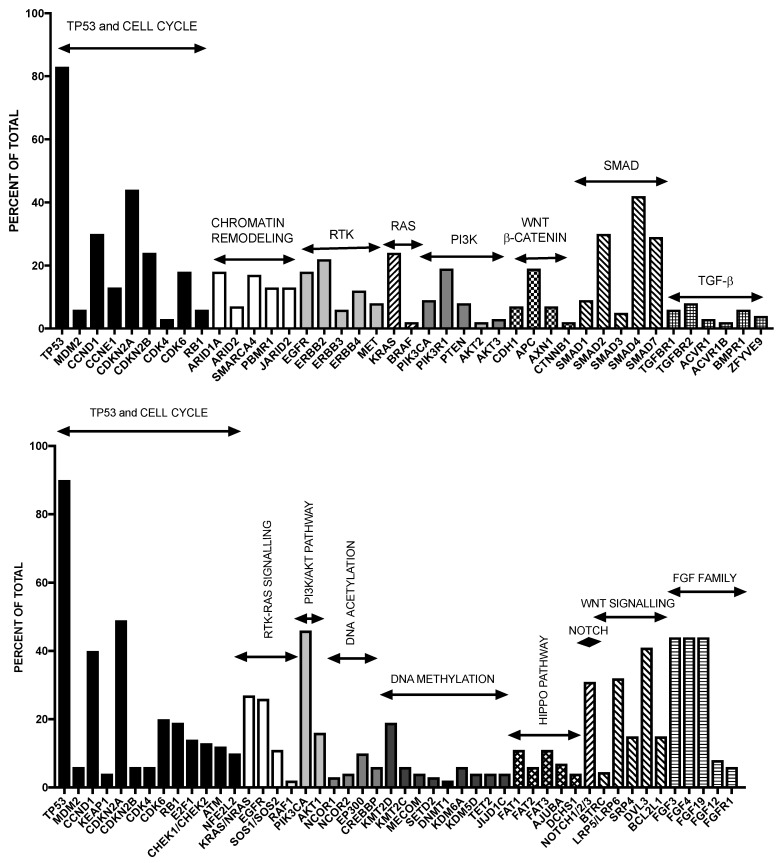
Frequency of the main genetic alterations observed in esophageal adenocarcinoma (EAC) (**top panel**) and in esophageal squamous carcinoma (ESCC) (**bottom panel**). The figure reports the cumulated frequency of both mutations and copy number alterations for the various genes indicated. The top panel is based on results reported by Dulak et al. [7] and Secrier et al. [9]. The bottom panel is based on data reported by Liu et al. [10], Song et al. [11], Gao et al. [12], Cheng et al. [13].

The molecular alterations occurring in esophageal cancer have recently been explored in detail. In these studies, emphasis was given on determining the differences existing in the two major esophageal subtypes [2,3,4]. TP53 point mutations represent the most frequent gene mutations occurring in about 50% of cases, these mutations being detectable both in EAC and ESCC; TP53 mutations are detectable also in early metaplastic precancerous lesions. Recently, techniques of exome and whole-genome sequencing have identified some recurrent driver genetic events occurring in esophageal adenocarcinoma and have also supported the existence of a considerable complexity of the genetic abnormalities [7]. This analysis provided evidence about the existence of a high frequency of mutations in this cancer, inferior only to those observed in melanoma and lung cancer [7]; this important finding suggests that these tumors are exposed to and derived from the effects of various damaging agents, supported also by the peculiar environment created by gastric reflux and chronic inflammation. The analysis of the genes more frequently mutated in EAC provided many key data on the molecular pathogenesis of this cancer. This analysis has shown that 26 genes are frequently and significantly mutated in esophageal cancer (Figure 1). The two most significant tumor suppressors mutated in EAC are TP53 (72% of cases) and p16/CDKN2A (12% of cases). In addition to these two genes, there are also other significantly mutated genes. Among them, two significantly mutated genes are ELMO1 and DOCK2, encoding dimerization partners and intracellular mediators of the Rho family; ELMO1 or DOCK2 are mutated in 17% of cases, and their mutation determines an enhancement of cellular motility and favors tumor invasion [7]. Other significantly mutated genes are represented by ARID1A, SMARCA4 and ARID2, pertaining to the family of chromatin-remodeling factors, together being mutated in about 20% of cases; mutations are also found in other chromatin-modifying enzymes, including JARID2 and PBRM1 [7]. Another remarkable mutated gene is SPG20, mutated in about 7% of EAC, encoding spartin, a protein involved in various cellular functions, including the endosomal trafficking of growth factor receptors. Finally, TLR4 mutations are observed in 6% of EACs [2]. It is of interest to note that if one considers both gene mutations and gene amplifications, 48% of esophageal cancers have a genomic alteration in a pathway that can be pharmacologically targeted: this is the case for PI3KCCA, EGFR, ERBB2 and MET, just to mention the most frequently altered [7]. These data have thus provided a road map for the identification of key somatic genetic events occurring during the development of EAC and to try to understand how these events act at molecular and cellular level. Nones and coworkers, using a combination of whole-genome sequencing and single-nucleotide polymorphism array profiling, showed that genomic catastrophes are frequent in EAC, with about 32% of these tumors exhibiting chromothriptic events: these events lead to oncogene amplification through chromothripsis-derived double-minute chromosome formation (MYC and MDM2) or breakage-fusion-bridge (KRAS, MDM2 and RFC3) [8]. The extreme genomic instability observed in EAC could be derived by somatic BRCA2 mutations [8].

A very recent study provided a detailed whole genome sequencing analysis of EACs with the molecular characterization of 129 cases, showing that EAC is a heterogeneous cancer dominated by copy number alterations with frequent large-scale rearrangements [9]. In fact, among the genes more frequently altered (i.e., in >10% of cases) many more were rearranged, amplified or deleted than were affected by point mutations or insertions/deletions [9]. Many recurrently rearranged genes were observed in EACs: SMYD3 (39%), RUNX1 (27%), CTNNA3 (22%), RBFOX1 (21%), CDKN2A/2B locus (18%), CDK14 (16%), fragile sites such as FHIT (95%) and WWOX (84%) [9]. Somatic mobile element insertions were also frequent at the level of relevant genes: ERBB4 (about 5%); CTNNA3 (5%), CTNNA2 (3%); CDH 18 (3%) and SOX5 (2%). Amplified genetic loci involved genes such as ERBB2, EGFR, RB1, GATA 4/6, CCND1 and MDM2, while deleted genetic loci involved genes such as CDKN2A, CDKN2B, CLDN22, several fragile sites [9]. The most frequent mutational events occurred at the level of TP53 (81%), ARID1A (17%), SMAD4 (16%), CDKN2A (15%), KCNQ3 (12%), CCDC 102B (9%) and CYP7B1 (7%) [14]. Importantly, large-scale genetic events are frequently observed in EACs: chromotripsis (30%), kataegis (31%) and complex rearrangement events (32%). This study showed also that Receptor Tyrosine Kinase Receptors (RTK) and their targets are frequently disrupted in EACs; particularly high-level amplifications are frequently observed for ERR2 (17%), EGFR (11%), MET and FGFR, with a global frequency of RTK amplifications corresponding to 43% [9]. In addition, genetic alterations are also frequent at the level of the downstream signaling pathways MAPK and PI3K [9]. These observations are important because indicate that only multiple kinase inhibitors may induce an efficient inhibitory effect on EAC cells [9]. Importantly, through the analysis of molecular signatures three distinct molecular subtypes with potential therapeutic relevance have been identified: (a) enrichment for BRCA signature with prevalent defects in the homologous recombinant pathway; (b) dominant T > G mutational pattern associated with a high mutational load and neoantigen burden; (c) C > A/T mutational pattern with evidence of an aging imprint [9]. These subtypes may represent a basis for a therapy selection of EAC patients [9].

The use of neo-adjuvant chemotherapy to shrink tumors before surgery offers the unique opportunity to compare the evolution of cancers that respond well and poorly to this treatment. Findlay and coworkers observed that the response of EAC genome to neo-adjuvant chemotherapy greatly varies: a group of poor responders EAC display only minor genomic changes following treatment; another group of patients displays multiple genetic driver mutations that variably increase or decrease in frequency following treatment, sometimes showing complete loss or gain; finally, a third group of patients was marked by clonal shifts, reminiscent of genetic bottlenecking [14]. In this context, the behavior of p53-mutant cells may be considered paradigmatic: some cancers retain their p53 mutation after treatment; other cancers harbor multiple single nucleotide variation or copy number alterations that can be lost, gained or change in their frequency after treatment; finally, in other cancers, p53 mutations can be lost in the absence of CNAs, since the mutant p53 resides in tumor cell clones that are lost as they pass through a genetic bottleneck [14]. Another study evaluated the genomic complexity of 8 EAC patients undergoing neo-adjuvant chemotherapy [15]. The existence of a high intra-tumor heterogeneity was associated with a poor response to the adjuvant treatment [15]. Noorani and coworkers have analyzed a large set of EACs pre- and post-chemotherapy (some matched and the majority not-matched). And reached the conclusion that they reveal no significant differences in the overall mutation rate, mutation signatures, specific recurrent point mutations or copy number events in respect to chemotherapy status [16]. This finding is not surprising in view of the well-known chemoresistance of EAC [16].

It is important to note that Ras mutations are rare in esophageal cancer. Initial studies have highlighted the low frequency (<5%) of K-Ras mutations in both esophageal adenocarcinomas and squamous carcinomas, while a high frequency (around 40%) was observed in colon cancer [17]. These investigations were extended also to N-Ras and BRAF that were found never mutated in esophageal cancers [17].

As stated above, observational studies have indicated that a number of factors, including chronic gastro-esophageal reflux, cigarette smoking, obesity and *Helicobacter pylori* Cag A seronegativity account for the large majority (about 75%–80%) of esophageal adenocarcinomas. However, there is evidence that in addition to the factors, also genetic factors play an important role in the genesis of esophageal adenocarcinoma and of its precursor lesions. Familial studies have suggested the existence of a common genetic background when a relative is affected by either chronic gastro-esophageal reflux or Barrett’s esophagus or esophageal adenocarcinoma (a 2–4-fold increased risk when a relative is affected); furthermore, twin studies have indicated a moderate heritability of gastro-esophageal reflux disease. Segregation analyses of many pedigrees of familial Barrett’s esophagus supports an incompletely dominant inheritance model with a polygenic component. These observations have stimulated the genesis of wide association genetic studies on Barrett’s disease. These studies have led to the identification of some genetic loci, associated with an increased risk of developing Barrett’s disease. The first study identified two regions associated with disease risk: (a) chromosome 6p21 involving the major histocompatibility locus; (b) chromosome 16q24, involving FOXF1, a gene involved in esophageal development and structure [18]. A more recent study identified three additional regions: (a) the first is localized at 19p13, involving CRTC1, a gene encoding CREB-regulated transcription co-activator; (b) the first is localized at 9q22, involving BARX1, a transcription factor playing a relevant role in esophageal specification; (c) the third is located at 3p14 near to the transcription factor FOXSP1, which regulates esophageal development [19]. Genome wide association studies have recently led to the identification of new genetic loci associated with an increased susceptibility to the development of Barrett’s esophagus and EAC. These loci mapped within or near the genes CFTR, M5RA, LINC00208, and BLK, KHDRBS2, TPPP and CEP72, TMOD1, SATB2, HTR3C and ABCG5 [20]. The locus identified near HTR3C and ABCG5 was specifically associated with EAC and may therefore represent a genetic marker for prediction of the transition from Barrett’s esophagus to EAC [20].

### 2.2. Molecular Abnormalities of Barrett’s Esophagus

The presence in some individuals who develop EAC of a premalignant lesion offers a unique opportunity for genetic studies aiming to elucidate the evolution of genetic alterations occurring during the development of esophageal cancer. Barrett’s esophagus is the premalignant condition associated with the development of EAC and its study and characterization at cellular and molecular level is essential for a better understanding of the mechanisms responsible for EAC development. At histological level, Barrett’s esophagus is characterized by the replacement of the normal squamous epithelium of distal esophagus with columnar epithelium. Barrett’s esophagus progresses to EAC through intermediate histological stages: Barrett’s esophagus, low-grade dysplasia (LGD), high-grade dysplasia (HGD), EAC. Three types of non-dysplastic Barrett’s esophagus have been reported: with gastric metaplasia and length <3 cm; with intestinal metaplasia and length <3 cm; with intestinal metaplasia and length >3 cm. Barrett’s esophagus confers an absolute risk of progression to EAC of about 0.5 per patient per year; LGD is associated with a progression risk to HGD or EAC of about 9%–13% per patient per year; finally, HGD has a 25% risk of progress to EAC [21].

Studies on the transition of Barrett’s esophagus to EAC have initially focused on the alterations of p16 and TP53 genes. According to these results, two models were proposed. One model proposed by Maley and coworkers suggests that an initial mutation (most commonly inactivation of p16) confers a selective advantage to a cell population and this mutation is present in most of cells of Barrett’s esophagus; the acquisition of additional mutations (i.e., inactivating TP53 mutations) give rise to cell clones able to expand across the Barrett’s lesion [22]. Leedham et al., have proposed a different model where multiple independent clones develop within the Barrett’s esophagus and their evolution is regulated through a process of clonal competition [23].

**Figure 2:**
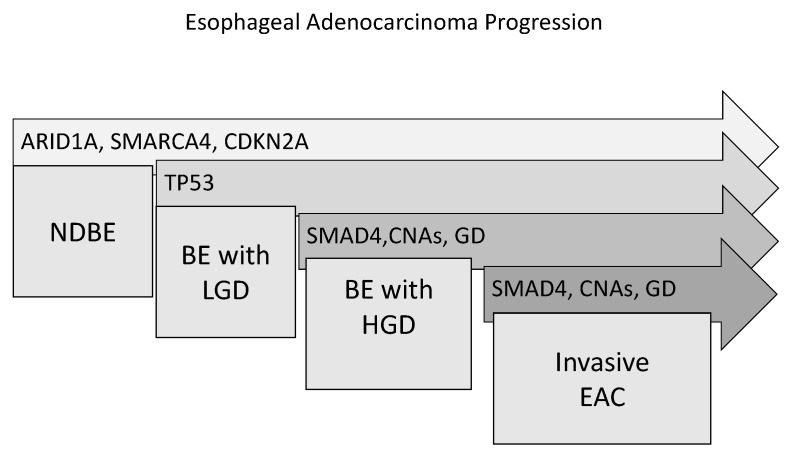
Model describing the progressive occurrence and accumulation of genetic alterations during the progression from non-dysplastic Barrett’s esophagus to invasive EAC, through the intermediate stages first of Barrett’s esophagus (BE) with low-grade dysplasia (LGD) and then BE with high-grade dysplasia (HGD). This model is based on results of studies reported by Weaver et al. [25] and Ross-Ines et al. [26].

In this context, Agrawal and coworkers have performed exome sequencing on 11 EAC samples and 2 samples of Barrett’s esophagus adjacent to the cancer; surprisingly, most of mutations were found to be present even in the Barrett’s esophagus samples [24]. More recently, Weaver et al., have analyzed in detail this important issue, providing important indications about the relative timing of mutations in esophageal carcinogenesis. Thus, using whole-genome sequencing and amplicon sequencing, these authors have identified recurrent genetic alterations occurring in 112 EACs and in transition tumor lesions: Barrett’s esophagus (66 cases) and high-grade dysplasia (43 cases). This study confirmed that the large majority of recurrently mutated genes in EAC were also mutated in Barrett’s esophagus [25]. Only TP53 and SMAD4 mutations occurred in a stage-specific manner, the first one being confined to high-grade dysplasia and the second-one to non-dysplastic Barrett’s esophagus (Figure 2). These findings clearly indicate that the few cancer driver mutations characterizing EC occur early during esophageal carcinogenesis [25]. These observations thus indicate that a complex mutational landscape may be even present at the level of a tissue with very low risk of malignant progression, such as Barrett’s esophagus never dysplastic, that has entirely a benign histo-pathological appearance [25]. The implications of these findings at the level of cancer biomarkers are that the large majority of recurrently mutated genes do not differentiate between the premalignant and malignant stages of disease and cannot be use as biomarkers of malignant progression.

The recent development of more sensitive sequencing techniques performed on small pieces of tumoral tissue allowed a more detailed comparison of the mutational spectrum of paired Barrett’s esophagus-EAC samples showing that surprisingly a relatively low degree of overlapping (<20%) was observed in most of cases; furthermore, the Barrett’s esophagus samples that had the best overlap with their paired EAC samples are those histologically classified as dysplastic [26]. These studies showed also that Barrett’s esophagus is highly mutated even in the absence of dysplasia (6.76 mutations/Mb, a mutation rate higher than for many other tumors at an advanced stage of development) [26]. Surprisingly, the paired analysis of mutations in Barrett’s esophagus and corresponding EAC showed that in more than 50% of cases, only a <20% correspondence in gene mutations was observed [26]. Cancer development is associated with a clear increase of copy number alterations: CNAs were rare in Barrett’s esophagus and their genomes are diploid, but frequent in EAC; the only frequent CNA observed in Barrett’s esophagus is 9pLOH [26]. TP53 mutations were less common in Barrett’s esophagus (39%) than in EAC (83%); similarly, other putative EAC driver genes, such as EYS, ARID1A and ABCB1, were mutated less commonly and are shared in only 28% of cases between paired Barrett’s and EAC samples [26]. Interestingly, the genetic analysis performed at the level of various areas of some Barrett’s esophagus lesions provided evidence about the existence of heterogeneous tumor clones, displaying each multiple genetic abnormality and some of these clones are responsible for progression to dysplastic lesions [26]. However, despite the differences in specific mutations, the general coding mutational context suggests a common causative mechanism underlying these two conditions [26].

**Figure 3:**
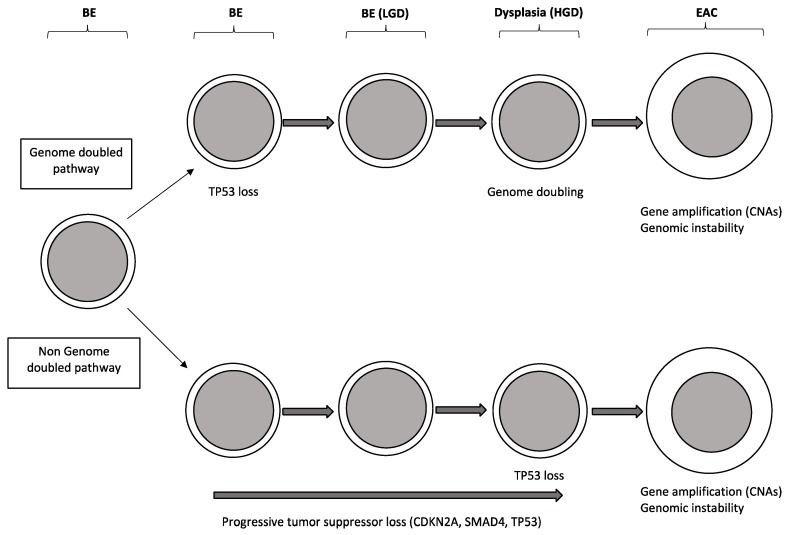
Schematic representation of two possible pathways of BE progression to EAC. The top model shows the tumor progression pathway involving genome doubling: this pathway implies the early occurrence of TP53; the genome doubling leads to genomic instability, oncogene amplification with frequent copy number alterations and aneuploidy. The bottom model shows the BE progression to EAC involving the gradual and progressive accumulation of tumor suppressor losses, followed by activation of oncogenes and development of genomic instability. Abbreviations: CAN: copy number alteration; BE: Barrett’s esophagus; LGD: low-grade dysplasia; HGD: high-grade dysplasia. This model is based on data reported by Stachler et al. [27].

Another study published in parallel carried out on 25 pairs of Barrett’s esophagus/EAC confirmed that the number of focal deletions and amplifications clearly increased during progression from Barrett’s esophagus without dysplasia, to Barrett’s esophagus with dysplasia and then to EAC [27]. Interestingly, exome analysis of EACs showed that the majority (about 62%) of these carcinomas emerged following genome doubling and that tumors with genome doubling exhibited different patterns of genomic alterations with more frequent oncogenic amplifications and less frequent inactivation of tumor suppressors, including CDKN2A [28] (Figure 3). Particularly, mutations of genes encoding chromatin modifiers, cell cycle regulators and TGF-beta pathway are more common in non-genome doubled EAC, compared to those with genome doubled [27]; in contrast, genome doubled EACs contain more frequent amplifications in cell cycle regulators and transcription factors [27] (Figure 3).

Li and coworkers have carried out a genetic analysis of a group of patients with Barrett’s esophagus studied in the time: the large majority (>95%) of these patients do not progress to EAC during their lifetimes [29]. The genomes of the non-progressors display some remarkable differences compared to progressors: the genomes of non-progressors usually had small localized deletions at the level of fragile sites and 9p(9pLOH), generating a low background of genetic diversity and remaining stable over a prolonged time; in contrast, progressors as they approach to EAC development, develop signs of chromosome instability with gene losses and gains, genomic heterogeneity, selection of somatic chromosome abnormalities and, finally, catastrophic genome doublings [29]. According to these findings it was proposed a model of disease evolution implying that non-progressor genomes remain stable in the time, whereas progressor genomes tend to evolution within few years, with increased genetic instability and acquisition of chromosome abnormalities [29]. Another recent report based on the use of multicolor fluorescence in situ hybridization on brush cytology specimens, reached the conclusion that the intrinsic genetic property of Barrett’s esophagus lesions represents the major determinant of their tendency to remain stable in the time or to progress to EAC: in fact, the observed data support a model where the risk of cancer evolution is mainly related to the acquisition of genetic instability early in pre-malignant lesion development [30].

In conclusion, the recent studies on the characterization of Barrett’s esophagus have provided evidence that this lesion is not simply a metaplastic tissue, but a pre-cancerous tissue, characterized by frequent somatic genetic alterations predisposing, in some cases, to cancer progression. Some of these molecular abnormalities can be used to predict the risk of Barrett’s esophagus progression to dysplasia first and then to cancer [31]. The molecular determinants responsible for the progression of Barrett’s esophagus to EAC remain, now, largely undetermined. The analysis of the data until now reported on the molecular characterization of Barrett’s esophagus, dysplastic lesions and EACs suggests a role for some selected genes in tumor progression: TP53, CDKN2A, CTNNB1, CDH1, GPX3 and NOX5 [32]. The actual view about the clonal evolution of Barrett’s esophagus suggests a model implying the sequential loss of tumor suppressor genes culminating in loss of TP53 and cancer development. In some cases, TP53 mutation can lead to cancer development more rapidly through chromosomal catastrophe or genome doubling and genetic instability.

### 2.3. Molecular Abnormalities of ESCC

Various genetic mutations have been identified in esophageal squamous cell cancers and many of them are associated with specific cellular pathways, such as cell cycle, apoptosis, DNA repair mechanisms, growth factor receptors. Recent studies have suggested that a major impact in this area could derive from comparative studies allowing a comparison of the mutational profile of ESCC, compared to EAC. In this context, particularly interesting are the results reported by Agrawal and coworkers [24]. These authors have reported the comparative exome sequencing of 11 EACs and 12 ESCCs [33] and observed that, while the mutational frequency at the level of the tumor suppressor TP53 was similar (73% in EAC and 92% in ESCC), NOTCH1 and NOTCH3 mutations were much more frequent among ESCC (33 and 25%, respectively) than EAC (0 and 9%, respectively) [24]. According to these findings these authors have explored NOTCH1 mutations in two larger groups ESCC patients, originating from two different geographical areas and observed a frequency of NOTCH1 mutations markedly higher in Northern American ESCCs (11 of 53 cases) than in Chinese ESCCs (1 of 48 cases) [24]. Now, the significance and the origin of this consistent geographic variation in the frequency of NOTCH1 mutations are largely unknown.

More recently, Chen and coworkers explored the occurrence and the possible functional implications of NOTCH 1 mutations and NOTCH pathway mutations in ESCC cancer development and progression [33]. These authors reported a frequency of NOTCH1 mutations in Chinese stage III ESCCs corresponding to 8% [33]. Interestingly, the frequency of NOTCH1 mutations was markedly higher for stage I ESCC patients, corresponding to 35% [33]. Mutations of the whole NOTCH pathway were observed in 55% of stage I tumors, versus 32% of stage III tumors [33]. According to these findings, it was concluded that NOTCH alterations are an early event in ESCC pathogenesis, playing an important role in early stages of tumor development [33].

An involvement of the NOTCH pathway in ESCC is also supported by recent studies showing a need for NOTCH signaling in esophageal epithelial homeostasis; the crosstalk of NOTCH1 and NOTCH3 is required for squamous differentiation [34]. Other studies have shown that NOTCH1 functions in two different ways in normal and pathological conditions: in normal conditions, NOTCH1 activity is sustained and mediates the balance between populations of the basal and differentiated esophageal cells; in pathological conditions, and particularly in precancerous and cancerous conditions, NOTCH1 expression is reduced and this hampers the normal epithelial differentiation, resulting in an immature epithelium [35]. In spite the not frequent NOTCH mutations in EAC, the NOTCH pathway is frequently activated in EAC due to impairment of the TGF-beta signaling. In fact, an impairment of the TGF-beta signaling pathway was frequently observed in Barrett’s metaplasia-dysplasia and esophageal carcinoma due to the frequent downmodulation of Smad4 related to various mechanisms, including promoter methylation, gene deletion and protein modification [36]. These findings were confirmed in another study showing the frequent loss of SMAD4 and β2 spectrin (β2SP) in esophageal adenocarcinoma inversely related to the expression of the NOTCH signaling components Hes-1 and Jagged1 [37]. A subsequent study showed that the loss of the TGF-β adapter β2SP was responsible for NOTCH signaling activation in esophageal cancer cells, inducing expression of NOTCH targets Sox9 and c-MYC and decreasing expression of TGFβ targets p21, p27 and E-Cadherin [38].

EGFR is overexpressed at protein level in about 50% of ESCCs and in about 30% of cases this gene is amplified [39]; interestingly, EGFR overexpression and TP53 mutations are very frequent in precancerous lesions and TP53 mutations are correlated with EGFR overexpression [39]. In line with these findings, EGFR overexpression and p53 mutations are necessary and sufficient to transform epithelial esophageal cells, leading to increased cell motility, anchorage independent growth, and tumor formation in nude mice [40]. EGFR mutations occur very rarely in EAC. EGFR was found to be expressed in 55%–60% of EACs: in 25% of cases EGFR seems to be overexpressed, while in only 4% of cases a EGFR gene amplification was observed [41]. EGFR was found to be overexpressed in 77% of ESCCs [42]. Importantly, elevated EGFR expression was found to be associated with higher pathologic tumor stages, lymph node metastasis and higher UICC stage and with reduced overall survival [42].

Genes involved in the control of cell-cycle are frequently altered in ESCCs. Thus, a loss of heterozigosity of the Rb gene, associated with low/absent Rb expression, was observed in >50% of ESCC samples [43]. P16INK4a expression is frequently reduced in ESCC and this is due to various mechanisms, including aberrant p16INK4a gene methylation observed in 62% of cases [44], loss of heterozygosity of the p16INK4a gene observed in 13% of cases [45], mutations of the p16INK4a gene observed in 6% of cases [44]. Cyclin D1 gene was amplified in 41% of ESCC patients; a significant proportion of these patients had a concomitant LOH of Rb [46] and these patients have a negative prognosis.

In initial studies mutations of the PI3KCA gene, which encodes the p110alpha catalytic subunit of PI3K have been reported in 2%–12% of ESCC patients. Recently, the occurrence and the prognostic impact of PI3KCA mutations was analyzed in many ESCC patients, showing that PI3KCA mutations were detected in 21% of patients and, compared with wild-type PI3KCA patients, these patients displayed a better prognosis, as analyzed in disease-free survival and overall survival [28]. PI3KCA gene is mutated in 6% of esophageal adenocarcinomas [47]. Additional studies have explored abnormalities of other members of the PI3K/AKT signaling pathway. Loss of PTEN expression was observed both in 25% of ESCCs [48] and 14% of EACs [42] and was a negative prognostic index for both these tumor types. Other studies have explored the activation of mTOR, an important effector of the PI3K signaling pathway. 25% of ESCCs exhibited overexpression of the mTOR and were associated to a poor survival [49]. mTOR overexpression was observed in about 20% of EAC patients and was associated with poor overall survival [50].

Another frequent genetic abnormality observed in ESCC is represented by the overexpression of the transcription factor SOX2. A copy gain number of SOX2 gene was observed in 15% of ESCC patients and SOX2 protein was overexpressed in 70% of ESCC tumors [51]. The increased SOX2 expression observed in ESCC seems to be relevant for the development of this tumor since: (a) SOX2 is mutated in esophageal malformations and its expression is required for normal esophageal squamous development; (b) SOX2 expression is required for proliferation and anchorage-independent growth of ESCC lines; (c) SOX2 cooperates with FGFR2 to induce squamous tumor formation in immortalized tracheobronchial epithelial cells [52]. In a recent study, the mechanisms through which SOX2 promotes ESCC cell proliferation have been explored: using a phosphoprotein array it was provided evidence that SOX2 activates AKT/mammalian target of rapamycin complex 1 (mTORC1) signaling, and through this mechanism promotes ESCC proliferation [53]. In line with this observation, in primary ESCCs a positive correlation was observed between SOX2 levels and AKT levels [53]. SOX2 expression was compared in ESCCs and in EACs showing that 85% of the former ones and 35% of the latter ones were positive [54].

Three recent studies have provided a global characterization of the molecular abnormalities occurring is squamous esophageal cancer. A first study by Lin and coworkers provided evidence about the recurrent mutation of TP53, PIK3CA, NOTCH1, FAT1, FAT2, ZNF750 and KTM2D genes in Chinese ESCC primary tumor samples [10]. These mutations were functionally relevant for the oncogenic process. The analysis of the biological pathways deregulated in ESCC indicated that RTK-MAPK-PI3K pathways, cell cycle and epigenetic regulatory mechanisms are frequently dysregulated by multiple molecular abnormalities in ESCC [10]. A second study, always carried out on Chinese ESCC patients reported a comprehensive genomic analysis on 158 tumor samples [11]. This analysis provided evidence about recurrent mutations at the level of: six well known tumor-associated genes, such as TP53, RB1, CDKN2A, PIK3CA, NOTCH1, NFE2L2; two not previously reported genes, such as ADAM29 and FAM135B; six histone regulator genes, such as MLL2 (KMTD2), MLL3 (KMT2C), ASH1L, SETD1B, CREBBP and EP300 [11]. Analysis of the pathway assessment indicated that the genetic abnormalities occurring in ESCC involve three pathways: Wnt, cell cycle and NOTCH [52]. A third study provided an overall view of the genetic landscape of ESCC: this analysis was based on the exome sequencing of 113 ESCCs. Importantly, the mutational profile of ESCC resembles mutational profiles of other squamous cell carcinomas, but differs significantly from that of EAC [11]. This fundamental analysis showed that: (a) genes involved in the regulation of apoptosis and cell cycle are mutated in virtually all cases (99%): TP53 (93%), CCND1 (33%), CDKN2A (20%), NFE2L2 (10%) and RB1 (9%); (b) histone regulatory genes are frequently mutated: KMTD2 (9%), KMT2C (6%), KDM6A (7%), EP300 (10%) and CREBBP (6%); (c) the Hippo pathway is frequently deregulated due to mutations in FAT1, FAT2, FAT3 or FAT4 (27%); (d) the NOTCH pathway is frequently deregulated by mutations inn NOTCH1, NOTCH2 or NOTCH3 (22%) or FBXW7 (5%) [12] (Figure 1).

Deletions and translocations are the dominant structural variation types observed in ESCCs, and 16% of these deletions were complex deletions. The structural variations frequently led to disruption of cancer-associated genes (e.g., CDKN2A and NOTCH1) with different mutational mechanisms. Moreover, Chromotripsis, kataegis, and breakage-fusion-bridge (BFB) were identified as contributing to locally miss-arranged chromosomes that occurred in 55% of ESCCs. These genomic catastrophes led to amplification of oncogene through chromotripsis-derived double-minute chromosome formation (e.g., FGFR1 and LETM2) or BFB-affected chromosomes (e.g., CCND1, EGFR, ERBB2, MMPs and MYC), with approximately 30% of ESCCs harboring BFB-derived CCND1 amplification [13]. Furthermore, analyses of copy-number alterations reveal high frequency of whole-genome duplication (WGD) and recurrent focal amplification of CDCA7 that might act as a potential oncogene in ESCC. These findings reveal molecular defects such as chromotripsis and BFB in malignant transformation of ESCCs [13].

The large majority of molecular studies on the characterization of genomic abnormalities of ESCCs have been carried out in Chinese patients. A recent study reported a wide exome sequencing and SNP array-based copy number analysis on 144 Japanese patients [55]. A high proportion of mutations in these patients were C to G/T substitutions with a flanking 5’ thymine (“APOBEC signature”). According to the mutational signatures, patients were subdivided into three subgroups, associated with environmental (drinking and smoking) and genetic (polymorphisms in ALDH2 and CYP2A6) factors [13]. Many ESCCs contained genetic alterations at the level of genes that regulate cell cycle (TP53 mutated in 93%; CCND1 amplified in 46%; CDKN2A mutated in 8% and deleted in 47%; FBXW7 mutated in 5%), epigenetic processes (MLL2 mutated in 18%; EP300 mutated in 8%; CREBBP mutated in 7.5%; TET2 mutated in 6%), NOTCH signaling pathway (NOTCH1 mutated in 18.5%; NOTCH3 mutated in 7.5%), WNT signaling pathway (FAT1 mutated in 14.5%; YAP1 amplified in 5.5%; AJUBA mutated in 4%), RTK and PI3K (PIK3CA mutated in 10.5%; EGFR amplified in 7%; ERBB2 amplified in 2.5%) [10]. In addition, frequent CNAs were observed at the level of TERT (amplification, 23%), PCDH (amplification 13%), LRP1B (deletion 21%), FOXA1 (amplification, 8%), FAM190A (deletion, 8%), HOXA cluster (amplification, 4%) [55]. The biochemical pathways most frequently deregulated in ESCC for the occurrence of genetic alterations are the cell cycle pathway (98%), epigenetic regulation pathway (59%), the NOTCH (33%) and RTK/PI3K pathway (32%) [55]. A comparison with published Chinese ESCC data indicates that the mutational spectrum is well conserved across these cohorts of patients [54]. ESCC is endemic also in regions of sub-Saharan Africa, where it is the third most common cancer. A recent study characterized at molecular level a population of sub-Saharan ESCC and showed in these patients, similar genetic aberrations as those reported in Asian and North American cohorts, including frequent mutations of TP53, CDKN2A, NFE2L2, CHEK2, NOTCH1, FAT1 and FBXW7 [55]. Analysis of mutation signatures showed the occurrence of three main signatures: a signature associated with aging, a signature associated with cytosine deaminase activity (APOBEC) and a third signature of unknown origin [56].

Recent studies of characterization of the genomic alterations occurring in ESCC have led to the identification of mutational signatures associated with peculiar pathogenic mechanisms. Thus, Chang and coworkers have performed a whole-genome sequencing analysis of DNA and RNA in 94 Chinese patients with ESCC [57]. Through this extensive analysis, they identified a mutational signature unique in ESCC, linker to alcohol intake and genetic variants in alcohol-metabolizing enzymes; the alcohol-driven ESCCs were characterized by a high frequency of mutations at the level of TP53, EP300, PTCH1, NOTCH3, TGFBR2 and ZNF750 [57]. These observations support at molecular level an important role of alcohol intake in ESCC etiology. Another recent study identified some mutations preferentially associated with ESCCs with lymph node metastases [58]. Metastatic ESCCs harbor frequent TP53, KMT2D, ZNF750 and IRF5 mutations; among these mutations, ZNF50 mutations were clearly more frequent in ESCC with lymph node metastasis than in those without metastasis [58].

**Figure 4:**
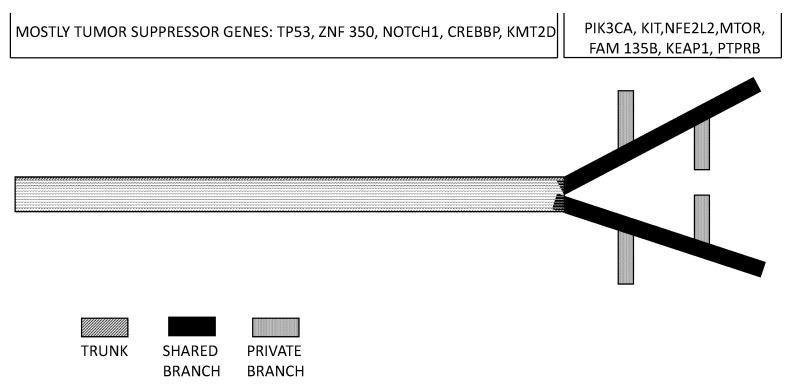
Intra-tumor heterogeneity of somatic mutations in ESCCs, as evaluated by multiregion whole-exome sequencing. The analysis of the mutational spectrum observed in different tumor regions allows the construction of a phylogenetic tree indicating the tumor evolution. In these phylogenetic trees, variable from one tumor to another, the trunk is defined as the initial clone, at the level of which are observed the initial genetic driver events responsible for tumor development; from this initial trunk, one or more branches are derived, maintaining some of the typical driver events observed in the trunk; finally, from the shared branches, one or more private branches may derive, characterized by the presence of mutations observed only in this tumor region. The result of this intra-tumor evolutionary process consists in an increase of tumor heterogeneity and by the development of a process generating multiple tumor regions with different biologic properties from a single initial tumor. On the top are reported the genes most frequently altered during the initial (trunk) and later (shared and private branches) phases of spatial-temporal development of ESCCs.

Surprisingly, tumor heterogeneity in ESCC is not well studied. Cao and coworkers profiled the mutations and copy number alterations that were identified in multiple regions within an ESCC from two patients [59]. The average mutational heterogeneity was 90% in all regions of each tumor in each patient. Phylogenetic analysis of the somatic mutation frequency of different tumor areas, supported the existence of multiple, genomically heterogeneous divergent clones evolving and co-existing within a primary ESCC and metastatic subclones originate from the migration and adaptation of an initially non-metastatic parental clone [59]. These observations are compatible with the existence of a highly evolving intra-tumor genomic heterogeneity in ESCCs [57]. This conclusion was reinforced by another recent study providing evidence, through the genetic analysis of various tumor regions of 13 ESCCs, about a great spatial intra-tumor heterogeneity. This analysis provided evidence about an average of bout 36% heterogeneous somatic mutations, with strong intra-tumor heterogeneity [60]. Phylogenetic tree construction, based on the results of multiregion whole-exome sequencing, allowed to establish that most of truncal and clonal driver mutations occurred in tumor-suppressor genes, such as TP53, KMT2D and ZNI750; in contrast, half of the driver mutations located on the branches of tumor phylogenetic trees involve oncogenes, such as PIK3CA, NFE2L2, KIT and mTOR [60] (Figure 4). According to these findings it was estimated that about 50% of driver mutations are branched or subclonal and therefore are a late event in tumor evolution (Figure 4). It is evident that targeting clonally dominant driver mutations (corresponding to early events) represents an optimal therapeutic strategy. It is also evident from this study that the evaluation of tumor genetic abnormalities based on a single biopsy determines an underestimation of tumor complexity and heterogeneity [60].

It is important to note that the incidence of ESCC is particularly high in China and it was estimated that approximately half of the world’s new ESCC cases each year occur in China. This finding has prompted specific studies aiming to identify some genetic factors present in this population that could facilitate the development of ESCC. The screening of large numbers of ESCC patients and of control healthy individuals allowed the identification of some polymorphic alleles as ESCC susceptibility alleles [61].

### 2.4. Comparison between EAC and ESCC

The comparative analysis of somatic copy number alterations in the adenocarcinomas of the esophagus, stomach and colon provided interesting data about the differential molecular mechanisms responsible for the genesis of these tumors. Thus, Dulak and coworkers observed a higher number of focal amplifications in the upper gastrointestinal adenocarcinomas (esophagus and stomach), compared to colon/rectal cancers [62]. To explain this finding, two hypotheses have been proposed: in esophageal and gastric cancers, the bile and acid injury may induce the generation of DNA strand breaks and thus contribute to high rate of somatic copy alterations; alternatively, distinct DNA reparative pathways are responsible for these differences [60]. Surprisingly, these focal amplifications in esophageal and gastric cancers are not accompanied by a concomitant increase of focal deletions [62]. Particularly interesting was the observation that the focal amplifications of receptor tyrosine kinases, including ERBB2, EGFR, MET, FGFR1 and FGFR2, are markedly more frequent in esophageal adenocarcinomas (42%) than in gastric (28%) and colorectal (14%) cancers [62].

Some recent studies have comparatively analyzed the genomic profiling of EAC and ESCC showing similarities, but also several remarkable differences. At the level of genetic alterations observed in single biochemical pathways, some pathways were similarly affected (cell cycle and epigenetic regulations); other pathways such as ERBB, RAS/RAF/MEK and TGF-β signaling were less mutated in ESCC than in EAC; finally, other pathways such as KEAP1/NRF2, NOTCH, FGF and PI3K/AKT/MTOR signaling were more mutated in ESCC than in EAC [61]. At the level of single genes, the most remarkable differences were observed for a set of genes (ERBB2, KRAS, SMAD4 and EGFR) less mutated in ESCC than in EAC, while another set of genes (PTEN, PIK3CA, CCND1, NFE2L2, NOTCH1, MLL2 and SOX2) is more mutated in ESCC than in EAC [63].

The Cancer Genome Atlas Research Network recently reported an integrated genomic characterization of esophageal carcinoma. Independent and integrated analysis of SNCA, DNA methylation, mRNA and miRNA expression profile allowed a clear separation between EAC and ESCC, thus indicating that they are really two distinct tumor entities. At the gene expression level, EAC showed increased E-cadherin signaling and upregulation of ARF6 and FOXA pathways, regulating E-Cadherin; E SCCs were characterized by upregulation of Wnt, syndecan and p63 pathways required for squamous cell differentiation [56]. SNCAs recurrent in EACs, but absent in ESCC, were amplifications of VEGFA, ERBB2, GATA6 and CCNE1 and deletion of SMAD4; in contrast, recurrent focal SNCAs in ESCCs included amplifications of SOX2, TERT, FGFR1, MDM2, NKX2-1 and deletion of RB1, VGLL4 and ATG7 [64]. The analysis of the mutational profile confirmed frequent mutations of TP53, NFE2L2, MLL2, ZNF750, NOTCH1 and TGFBR2 in ESCC, while in EAC frequent are the mutations of TP53, CDKN2A, ARID1A, SMAD4 and ERBB2 [64]. An overview of the various genetic abnormalities occurring in esophageal cancers showed that: abnormalities of cell cycle-related genes are highly frequent (90% in ESCC and 86% in EAC), followed by RTK abnormalities (59% in ESCC and 76% in EAC), cell differentiation (57% in ESCC and 42% in EAC), embryonic development (38% in ESCC and 53% in EAC) and chromatin remodeling (36% in ESCC and 22% in EAC). Importantly, the integrated analysis of genetic abnormalities occurring in ESCC allowed a new classification in three subtypes: ESCC1 was characterized by frequent alterations of the NRF2 pathway, involved in the adaptation to oxidative stress [with frequent alterations of the NRF2 (NFE2L2), KEAP1, CUL3 and ATG7 genes] and of SOX2 and TP63 (gene amplification); ESCC2 was characterized by frequent mutations of NOTCH1 or ZNF750, inactivating alterations of KDM6A or KDM2D, amplifications of CDK6 and inactivation of PTEN or PIK3R1; ESCC3 tumors do not display deregulation of cell cycle genes, more rarely (25%) had TP53 mutations and many genetic alterations are related to the activation of the PI3K pathway [56]. Finally, the comparison of the molecular abnormalities of esophageal cancers with those of tumors occurring in anatomic regions nearest to esophagus showed that EAC resembles gastric adenocarcinoma, while ESCC mostly resembles head and neck squamous carcinoma [64].

### 2.5. Gene Expression Studies

In parallel to studies aiming to define genetic alterations present in esophageal cancers, other studies attempted to identify gene expression profiling in EAC and ESCC allowing to identify patient’s subgroups with different prognostic significance and predictive of response to therapy. However, the numerous studies until now performed were heterogeneous in their methodology and in their results and have been unable to define a repeatedly identified gene signature with clinical relevance [65]. In spite the important limitations, the available evidences indicate that gene signatures observed in esophageal cancer patients are of prognostic value for clinical outcomes and may represent a precious tool for selecting optimized therapy for the single patient [65]. Thus, several studies have identified in EAC gene signature profiling correlating with patient outcome: one study reported a 4-gene signature (DCK, PAPSS2 and SIRT2 underexpression, associated with TRIM44 overexpression) associated with a reduced survival [66]; a second study identified a 2-gene signature (combined overexpression of SPARC and SPP1) predicting poor survival [67]; a third study identified a 4-gene signature (EGFR, MTMR9, NEIL2 and WT1) able to stratify EAC patients in 5 survival groups [68]. In ESCC only one study reported a link between gene signature and patient’s survival: thus, the overexpression of CTNN was associated with a shorter survival [69]. Other studies have supported the identification of an association between some gene signatures and patient’s response to chemo-radiotherapy: thus, Ephrin B3 overexpression correlated with response to therapy in EAC patients [70]; a 5-gene signature (PERP underexpression and DAD1, PRDX6, SELPINB6 and SRF overexpression) predicted non-responders compared to responders [71]; a 5-gene signature (under-expression of EPB41L3, NMES1, RPNC1, STAT5B and RTKN overexpression) identify responders from non-responders in both EAC and ESCC patients [72]; a 3-gene signature (PERP, S100A2 and SPRR3 overexpression) characterized complete responders to chemo-radiotherapy in both EAC and ESCC patients [73].

Some single nucleotide polymorphisms consistently increase the risk of developing ESCC. Genome-wide association studies have identified two SNPs, rs671 in ALDH2 on 4q23 and rs1229984 in ALDH1B on 12q24 that are clearly associated with the risk of developing ESCC in a manner related with alcohol drinking and tobacco smoking status [74,75]. Chang et al., showed that the frequency of a peculiar mutation profile, called signature E4, was significantly higher in ESCC from drinkers with the risk ALDH2 genotype than in ESCC from drinkers with the non-risk genotype [57].

Recent gene expression studies have suggested new classification criteria for ESCC. Thus, Xiong and coworkers, using the specific 185-gene signature generated by unsupervised consensus clustering of gene expression data, defined four subtypes of ESCC: tumors with high metastasis, associated with EMT (epithelial to mesenchymal transition), poor differentiation, active MAPK4/JNK signaling pathway and poor prognosis; tumors with high chromosomal instability and high expression of MYC-regulated targets; well differentiated tumors, with less aggressive invasivity and better prognosis; a group of tumors with intermediate properties and moderate prognosis [76].

Finally, a recent study compared the gene expression profiles of the two major histological subtypes of solid tumors (adenocarcinomas and squamous cell carcinomas) across organs, with a focus on esophagus, lung and uterine cervix, showing that histology-related differences accounted for a more consistent degree of inherent molecular variation in the tumors that did the of origin [77]. Interestingly, this study underlined some genes, such as IGF2BP1, as a possible common driver of adenocarcinomas, and Liver X receptor activation, as a pathway upregulated in adenocarcinomas and downregulated in squamous cancers [77].

## 3. Normal Esophageal Stem Cells

The esophagus is derived from the anterior portion of the foregut, a structure giving rise also to trachea, lung and stomach. Multiple signaling systems (particularly Bone Morphogenetic Proteins, BMPs) and transcription factors (SOX2) are required for esophagus development. These factors play an essential role in the induction of differentiation of primitive esophageal epithelium undergoing a transition from a simple columnar to stratified squamous epithelium [78].

The normal esophageal epithelium exhibits three compartments: (i) a superficial squamous cell compartment; (ii) a differentiating suprabasal cell compartment, containing proliferating cells at various stages of differentiation; (iii) a proliferative basal cell compartment, in which stem cells and proliferating progenitors are in contact with the basement membrane. Basal cells progressively differentiate and migrate in an outward direction to the lumen and move from initial proliferative stages to terminal non-proliferating differentiation stages. It is unclear whether all basal cells have an equal stem cell capacity of self-renew or whether the basal cells are organized according to a hierarchical pattern, in a stem cell-transit amplifying cell population.

It is important to recall that the esophagus possesses submucosal glands composed by a single mucus-producing acinus that continues into developed ducts opening onto the surface epithelium. Most of these ducts is composed by columnar epithelium and the last distal, near to the surface, by squamous epithelium. Barrett’s metaplasia consists in the replacement of the normal squamous epithelium with a columnar-lined, mucus-secreting, epithelium.

Before analyzing the few data available about the cancer stem cells of esophageal cancer, it is important to briefly discuss the problem of the identification of normal esophageal stem cells. Esophageal epithelium consists of layers of keratinocytes and lacks structures such as crypts, which at the level of the intestinal epithelium form stem cell niches. In this epithelium, the proliferation is confined at the level of the basal layer. Various studies have provided evidence about the existence in the basal layer of murine esophageal epithelium of cells exhibiting properties of stem cells. Kalabis and coworkers, using Hoechst dye extrusion and BrdU label-retaining assays identified in mice a putative stem cell population localized at the level of the basal layer [79]. The self-renewal properties of these cells were evaluated through clonogenic assay and organotypic culture models and their epithelial in vivo reconstitution capacity was tested in direct esophageal epithelial injury model [80]. A recent report by Doupé and coworkers provided more clear and direct evidence that a single progenitor population present at the level of the basal lamina switches its behavior to maintain and to repair esophageal epithelium. Under normal, steady-state conditions, the esophageal epithelium is maintained by a single population of cells that divide stochastically to generate both proliferating and differentiating daughter cells; however, following wounding, stem cells receive signals allowing their switch to produce an excess of proliferating daughter cells, specialized to the reparation of the wound [80]. This mechanism of fate switching is important because it allows a single esophageal stem cell population both to maintain and to repair tissue, without a need for a reservoir of slow-cycling stem cell pool [80]. Other more recent studies have confirmed the presence of a stem cell population in the murine esophageal epithelium. This population corresponds to a small subpopulation of basal cells; in contrast, most of basal cells act as dividing transit-amplifying cell populations [81]. This stem cell population can be identified according to some markers (SOX2, CD73), while transit-amplifying cells are negative for these markers (CD73^−^) [82]. The activity of the keratin 15 (Krt15) promoter identifies a long-lived population of basal cells in the mouse esophagus capable of generating all states of squamous lineage commitments [83]. Genetic ablation of Krt15^+^ cells results in reduced proliferation and epithelial atrophy [83].

The study of human esophageal stem cells remains problematic and very few reports have addressed this important issue. The epithelial basal layer of the human esophagus consists of two distinct zones, one overlying the papillae of the supporting connective tissue and the other covering the interpapillary zone. Proliferating cells are rare in the interpapillary zone, where rare putative stem cells undergo asymmetric divisions, giving rise to one basal daughter and one suprabasal, differentiated daughter [82]. Okumura and coworkers have identified in the human squamous esophageal epithelium a keratinocyte stem/progenitor cell characterized by the expression of the p75^NTR^, the low-affinity neutrophin receptor: the cells positive for this receptor are slowly cycling both in vitro and in vivo, but when grown in vitro generate a population of highly proliferating beta1-integrin positive cells that give rise to all distinguishable keratinocyte subsets [84]. However, a recent report provided evidence about the existence in normal human esophagus of slowly cycling adult stem cells by tracking 5-iodo-2’-deoxyuridine (Idu) label-retaining cells (LRCs). To do this interesting study, four patients undergoing esophagectomy for various pathologies, received intravenous infusion of IdU; tissues were collected at various days after infusion, from regions of healthy esophagus, Barrett’s esophagus, esophageal dysplasia and adenocarcinoma [85]. The main results of this study were that significant numbers of LRCs were found in the papillae of the basal layer of the esophageal squamous epithelium, in the base of the glands of the Barrett’s esophagus, but not in esophageal dysplasia and in adenocarcinoma [82]. These LRCs are of epithelial origin, but do not express markers for goblet cells, neuroendocrine cells or Paneth cells; these cells are adponed to a population of proliferating cells [85].

Using 3D imaging, coupled with staining for a range of cell lineage markers, Barbera and coworkers have investigated the patterns of proliferation and mitosis in human esophageal epithelium. Using this approach several interesting conclusions have been reached: (i) the most quiescent cells expressing putative stem cell markers are located at the tip of the papillae; (ii) asymmetric divisions, which are a typical hallmark of stem cells, are not restricted to a specific cell compartment; (iii) cells at various stages of differentiation sorted according to the expression of progenitor cell markers have equal capacity for self-renewal and ability to reconstitute a squamous 3D architecture in vitro [86].

The factors controlling the proliferation and differentiation of esophageal stem cells are poorly known. In this context, a recent study provided evidence that NOTCH signaling is essential for the differentiation of these cells. In fact, the introduction of a NOTCH mutant causing inhibition of the NOTCH signaling at the level of the esophageal basal cells promotes a suppression of differentiative mitosis of these cells, thus inducing their expansion as undifferentiated cells and the formation of clones expanding and progressively replacing the entire epithelium [87]. Analysis of gene expression in mutant cells reveals alterations in the expression of genes implicated in keratinocyte differentiation: thus, the stress-induced keratin Krt6 is strongly induced in mutant cells. In contrast, Sox9 is downmodulated in mutant cells [87].

## 4. Cellular Origin of Barrett’s Esophagus

The frequency of progression of Barrett’s esophagus to EAC is low. Two recent studies have provided an accurate evaluation of the frequency of malignant progression of Barrett’s esophagus: in a first study, 11,028 patients with Barrett’s esophagus have been analyzed in the time for 5.2 years showing an incidence rate of adenocarcinoma of 12 cases per 1000 per year, which corresponds to a risk of developing EAC 11.3 higher than in the general population (the risk of malignant progression was higher in patients with Barrett’s esophagus with dysplasia) [88]; in a second study carried out in Northern Ireland on 8522 patients with Barrett’s esophagus, the combined incidence of EAC, gastric cancer cardia and high-grade dysplasia was 0.22% (this frequency was higher, 0.38%, in patients with Barrett’s esophagus with specialized intestinal metaplasia) [89]. Both these studies unequivocally show that the risk of malignant progression among patients with Barrett’s esophagus is low.

As stated above, the large majority (>95%) of subjects with Barrett’s esophagus do not progress to esophageal adenocarcinoma during their lifetimes, while a minority of these patients develop esophageal adenocarcinoma. Using a longitudinal case-cohort study, somatic chromosomal alterations have been explored in two groups of Barrett’s esophagus patients, one not-progressing and the other progressing to EAC [90]. The genomes of the non progressors usually display small localized deletions involving fragile chromosome sites and 9p loss, generating a small genetic diversity that remained constant over time; in contrast, the genomes of progressors developed chromosome instability with gains and losses, resulting in a consistent genomic diversity and in numerous somatic chromosomal alterations (these changes occur within four years of EA diagnosis) [90].

Animal models to study Barrett’s metaplasia were mainly based on a rat model involving esophago-jejunostomy that induces gastro-duodenal reflux. Reflux injury in the esophagus results from chronic inflammation, associated with production of cytokines, such as IL-1beta, IL-6 and IL-8, all contributing to metaplastic and dysplastic conversion. These observations have represented the basis for the development of a model of Barrett’s-like metaplasia based on the overexpression of IL-1beta in the mouse esophagus [91]. IL-1beta overexpression in the esophagus of these mice caused esophagitis, Barrett’s-like metaplasia and neoplasia [91]. In this mouse model, exposure of the esophagus to bile acids accelerates the development of Barrett-like metaplasia and dysplasia, through a mechanism involving a strong activation of the NOTCH signaling pathway [91]. The analysis of this mouse model allowed also to explore the cell progenitor involved in the development of Barrett metaplasia, providing evidence in favor of a possible involvement of gastric cardia progenitor cells [92]. The analysis of this mouse model of Barrett’s esophagus provided evidence that increasing stem cell marker LGR5 and niche cell marker DCLK1 and decreasing differentiation marker (secretory mucus cells, TFF2^+^ cells) correlated with a high tumor score [92]. In a subsequent study, it was tested the applicability of these markers for human Barrett’s esophagus, particularly for distinguishing patients with Barrett’s esophagus with no evidence of dysplasia from those with dysplasia [92]. Low levels of TFF2 supported the best discrimination between nondysplastic Barrett’s esophagus and Barrett’s esophagus with cancer, followed by high levels of DCLK1, low goblet ratio and high LGR5 expression [92]. These findings support a risk prediction model of cancer progression of Barrett’s esophagus.

The inflammatory cytokines revealed at the level of the inflammatory esophagus resulted in a stimulation for the migration of cardia progenitor cells, including LGR5^+^ cells, and their metaplastic descendants into the esophagus. The role of inflammatory cytokines in the development of Barrett’s esophagus and in its progression to EAC is supported also by studies on patients with Barrett’s esophagus. These studies were based on the use of non-steroid anti-inflammatory drugs (NSAIDs) as agents capable of reducing the risk of cancer development. The action mechanism of these agents is largely unknown, but it is reasonable to assume that these agents act by lowering the development of somatic genetic abnormalities. This hypothesis was directly evaluated by studying the effect of NSAIDs administration on the number of somatic genetic abnormalities in patients with Barrett’s esophagus: the evaluation in the time of these patients showed that those receiving NSAIDs developed significantly less somatic genetic abnormalities than those not treated with these drugs [93].

A recent study provided some evidence about the progenitor responsible for the development of metaplasia and for a novel mechanism responsible for the generation of metaplasia, apparently not necessarily involving genetic mutations. In fact, according to the studies carried out in a peculiar murine system it was provided evidence that the earliest events that lead to metaplasia development do not depend on genetic changes, but rather on competition between cell lineages for access to basement membrane, a site essential for cell proliferation [94]. This conclusion was based on the study of p63-deficient mice, which lack squamous epithelia. The p63 null embryo rapidly develops intestine-like metaplasia, with gene expression profiles like those observed in Barrett’s metaplasia [94]. According to these findings, it is tempting to suggest that a similar cellular mechanism could occur in the early phases of esophageal metaplasia development. The contribution of inflammation at early times could consist in altering the competitive status between endogenous cell populations and “opportunistic” cell populations. At later times of metaplasia development, the role of inflammation is more clear contributing to the development of proliferation-related mutations and of epigenetic basis the becomes the essential molecular mechanism for the development of a clonal selection condition which determines disease progression. An additional fundamental finding of this study is related to the identification of a putative progenitor of esophageal metaplasia. In fact, cell tracking experiments carried out in the p63-null mouse model identified as metaplasia-progenitor a unique population of embryonic epithelial cells persisting in adult mice and humans and residing at the level of squamo-columnar junction [94]. Following damage of the squamous epithelium, these embryonic cells migrate toward adjacent squamous cells according to a process mimicking the development of Barrett’s metaplasia [94]. The migration of these residual embryonic cells at the level of the squamous esophageal epithelium would represent the initial step in Barrett’s metaplasia development.

Thus, according to these studies, it must be concluded that the conversion of normal esophageal epithelium to intestinal metaplasia is not well understood and it unclear whether the metaplastic epithelium is derived from the normal esophageal epithelium or arises from gastric cardia stem cells or from remnant embryonic epithelial cells that persist at the gastro-esophageal junction.

Studies on the origin of human Barrett’s metaplasia have shown a more complex pattern of cellular origin compared to those described through the study of animal models. Leedham and coworkers have provided evidence, through the analysis of tumor suppressor loss of heterozygosity at the level of individual crypts, that the ducts could represent the source of the Barrett’s metaplasia, as supported by the finding that normal squamous ducts contained the same somatic mutation as the contiguous metaplastic epithelium [95]. This and other studies have addressed also the problem of the cellular origin of neo-squamous islands that can arise in the context of Barrett’s tissue after acid suppression or endoscopic ablative therapy. Paulson and coworkers have demonstrated that these islands of squamous tissue were usually genetically wild-type, despite being surrounded by mutated Barrett’s tissue [96]. This finding suggests a stem cell origin within the Barrett’s metaplasia different from that observed in the neo-squamous tissue [96]. Recently, mitochondrial DNA mutations have been used as markers of clonal expansion to investigate the stem cell architecture of the normal esophagus and of the glands in Barrett’s metaplasia. These studies allowed to show that the normal squamous epithelium contains clonal patches of variable size, thus derived from the differentiation of a single stem cell; in contrast, a single esophageal gland duct contains multiple cells, mutated and not for mitochondrial DNA, thus indicating that seemingly they derive from multiple stem cells [97]. At the level of metaplastic Barrett’s glands two types of patterns were observed: all the cells of the gland are of clonal origin; the cells composing the gland are of multiple origin [98]. These two patterns underline the process of clonal evolution of the Barrett’s metaplasia, with the progressive development of a dominating clone [98].

The factors that favor the metaplastic change of esophageal epithelium into columnar epithelium are largely unknown. Several signaling pathways and transcription factors have been potentially involved in the pathogenesis of Barrett’s metaplasia, for their role in the development and homeostasis of the normal gastro-intestinal system. A squamous epithelium may be induced to develop a columnar phenotype through either the decreased expression of squamous cell transcription factors (such as p61 or SOX2) or increased expression of columnar cell transcription factors (such and CDX1, CDX2, MATH1 or SOX9). This hypothesis is supported by the observation that decreased expression of squamous cell transcription factors and increased expression of columnar cell transcription factors is observed at the level of Barrett’s esophagus tissue [97]. During this process, in humans it was suggested that two stages can be observed: a first one characterized by a non-specialized columnar metaplasia, preceding the development of the second one characterized by intestinal metaplasia [99]. Other recent studies suggest a key role for Hedgehog (HH) signaling pathway in BE development. The HH signaling is active in early embryonic life when the esophagus is lined by columnar epithelium, but when the embryonic columnar lining epithelium differentiates into stratified squamous epithelium, HH signaling is repressed. Gastro-esophageal reflux reactivates HH signaling in mouse and HH pathway was found to be activated at the level of BE tissue [99]. The metaplastic effect of HH on squamous esophageal epithelium is mediated by upregulation of FOXA2: FOXA2 was found to be overexpressed in Barrett’s metaplasia, dysplasia and EOC, while it was not expressed in ESCC [100]. FOXA2 was responsible for the induction of some genes, such as MUC2, well expressed in BE [100]. Using animal models and in vitro assays, it was provided evidence that non-specialized metaplasia is a precursor of intestinal metaplasia and that Bone Morphogenetic Protein 4 (BMP4) is an important contributor to this process through the induction of a pSMAD/CDX2 complex [101].

The origin of Barrett’s esophagus was recently explored by a combination of molecular studies and by the development of a new technology of isolation of intestinal stem cells derived from various areas of the human intestine. This new methodology of cultivation of human intestinal stem cells supports the maintenance of human intestinal stem cells in a highly clonogenic, ground state form [102]; this methodology is largely based on the development of media containing complex combinations of growth factors and regulators of TGF-β/BMP, Wnt/β-catenin, EGF, IGF and Notch pathways [102]. These resident stem cells possess a well-defined epigenetic program of cell differentiation, allowing their specialized commitment to various intestinal areas, a property stably maintained during the in vitro culture of these cells [102]. Yamamoto and coworkers have used this methodology to study intestinal clonogenic cells isolated from Barrett’s patients and have compared these cells to intestinal stem cells isolated from normal esophagus and stomach [103]. Clonogenic cells isolated from Barrett’s esophagus are distinct from those isolated from normal esophagus and stomach, in that these cells differentiate to columnar epithelium with path gnomic Alcian blue-goblet cells, while clonogenic cells of normal esophagus generate a progeny of squamous cells [103]. The analysis of the histological pattern, membrane and biochemical markers provided evidence that Barrett’s stem cells are distinct from stomach and intestine stem cells [94]. The analysis of the transformation capacities of Barrett’s and esophageal cancer stem cells support the conclusion that the two different forms of esophageal cancer arise from two different types of stem cells that are committed to esophageal epithelium (ESCC) and Barrett’s intestinal metaplasia (EAC) [103]. The genomic analysis of Barrett’s stem cells shows a broad and variable mutational spectrum: 25% of cases do not show typical cancer-related genomic changes; most of cases display patterns of deletions like those observed in EAC, but gene-amplifications were absent; importantly, the cases showing signs of low-grade dysplasia, exhibit p53 mutations or amplifications of proto-oncogenes and RTK [103].

In conclusion, the studies carried out to define the cellular origin of Barrett’s esophagus have led to various hypotheses, indicating different cellular sources: progenitor cells located in the esophagus (either within the squamous epithelium or in submucosal glands), columnar progenitors located in the squamo-columnar junction or in gastric cardia, migrating in the esophagus. Whatever the cellular origin is, the cells involved in the formation of Barrett’s esophagus must undergo a complex process of molecular reprogramming to generate a specialized intestinal metaplastic tissue. Some transcription factors play an essential role in the transdifferentiation/transcommitment processes. 

## 5. Esophageal Cancer Stem Cells

Few studies have characterized cancer stem cells at the level of esophageal cancers (Table 1). In this context, initial studies have been based on the isolation and characterization of cancer stem cells from esophageal cancer cell lines [104,105,106]. These studies have shown that cell subpopulations identified according to CD44 [104] or p75 neutrophin receptor (p75^NTR^ or CD271) [105,106] display properties of cancer stem cells and this conclusion is based on the capacity of these cells to initiate tumor, self-renew, and resist to standard chemotherapy. The discovery that CD44^+^ cells isolated from ESCC cell lines display properties of cancer stem cells greatly stimulated the study of CD44 expression at the level of both normal and tumor esophageal tissues showing that: (a) in normal esophageal tissue CD44 expression was limited at the level of the basal compartment containing putative stem cells; (b) 70% of ESCC specimens exhibited a pronounced expression of CD44, with various patterns of positivity [105]. Interestingly, induction of the differentiation of ESCC using retinoic acid was accompanied by a marked reduction of CD44 expression [107].

A more recent study showed that ICAM-1 is a potential membrane stem cell marker in ERSCC cancer cell lines. ICAM-1^+^ cells isolated from these cell lines have increased tumorigenic activity and are more chemoresistant. Interestingly, knockdown of ICAM-1 expression in esophageal cancer cell lines determines a concomitant upmodulation of CD44 expression; the same applies for CD44 in that CD44 downmodulation induces a concomitant ICAM-2 upmodulation [107]. According to these findings, it was proposed that an efficient targeting of ESCC cancer stem cells should involve dual targeting of both ICAM-1 and CD44 [107].

Concerning the identification of p75^NTR^ as a marker of tumor-initiating cells it is important to note that initial studies have shown that the low-affinity neurotrophin receptor p75 (NTR) characterizes human keratinocyte stem cells in vitro [84]. P75^NTR^ was expressed at the level of cellular elements present in the basal layer of the normal esophageal tissue [84]; p75^NTR^ expression was increased in ECs and the pattern of positivity at tissue level varied according to the degree of tumor differentiation [84].

A recent study showed that CD90 is commonly and moderately overexpressed in ESCC clinical specimens [103]. These findings prompted to analyze the expression of CD90 at cellular level using flow cytometry: CD90 was expressed in normal and premalignant dysplastic samples at the level of a minority of cells, ranging from 0% to 1.4%; CD90 expression was significantly higher in freshly isolated ESCC clinical samples, the percentage of positive cells ranging from 2.4% to 10% [108]. CD90^+^ cells were isolated from these tumor specimens and were shown to exhibit an enhanced capacity to initiate tumor formation into immunodeficient mice, self-renew, differentiate and are resistant to standard cytotoxic drugs [108]; furthermore, CD90^+^ cells were shown to possess a high metastatic potential in vivo [108]. The metastatic potential of these cells is related to the Ets-1 mediated expression of a matrix metalloprotease and a deregulated EMT phenotype [108].

A recent study showed that integrin α7 (ITGA7) is a functional marker of ESCC cancer stem cells. The frequency of ITGA7^+^ cells is significantly associated with poor prognosis, poor differentiation and lymph node metastasis [109]. ITGA7^+^ cells display enhanced stemness features, associated with enhanced self-renewal capacities and resistance to chemotherapy. At functional level, ITGA7 regulates cancer stem cell properties through FAK-mediated signaling pathways triggered by ligand-integrin interaction [109].

Another cell surface marker potentially identifying esophageal cancer stem cells is ABCG2, a member of group G in the ATP-binding cassette (ABC) transporter family. ABCG2 is expressed in the apical membrane of esophageal epithelial cells. ABCG2 contributes to the efflux of many chemotherapeutic drugs and is expressed together with vacuolar-H^+^-ATPase (playing a key role in the control of intracellular pH); both these transporters are over-expressed in ESCC and their expression was associated with pathological grade [110]. ABCG2 expression is upregulated by cigarette smoking and is inhibited by the drug mithramycin: remarkably, this drug decreases the proliferation and the tumorigenicity of esophageal cancer cells [111].

Other studies have further attempted to characterize populations of cells enriched in CSCs isolated from esophageal cancer cell lines. In this context, Zhang and coworkers have isolated tumorspheres from the Eca109 human EC cell line and have shown that these cells are enriched in Aldehyde Dehydrogenase positive cells and expressed various stem cell markers, such as Nanog and Oct4 [112]. A particularly high ALDH1 expression was reported in tumor-spheres isolated from esophageal cancer cell lines using a 3D-cell culture system [113].

Yang and coworkers confirmed the stem-like properties of ALDH1A^+^ cells isolated from the EC109 cell line [114]. Taking advantage on this observation, these authors have explored ALH1A expression at the level of tumor specimens obtained from 165 patients, showing that the level of ALDH1 positivity was associated with various tumor parameters related to the invasiveness and metastatic properties of ESCC and with poor prognosis [114]. Finally, they showed also that ALDHA1^+^ cells display molecular properties indicating an epithelial-mesenchymal transition [114].

These conclusions were confirmed in additional studies performed in EAC patients. Thus, Ajani and coworkers showed that ALDH1 expression correlated with the presence of CSCs in EAC patients and predicted complete tumor responses (CTRs): thus, patients with CTRs exhibited very low ALDH1 levels, while patients resistant to chemo-radiotherapy treatment display high ALDH1 levels and had higher incidence of tumor relapse and cancer-related deaths [115]. Furthermore, Honjo et al., provided evidence that ALDH1-positive cells are observed in both ESSCs and EACs and that the drug metformin targets these ALDH1^+^ CSCs [116]; in fact, this drug was able to inhibit the growth of these cells, to induce apoptosis and to sensitize to the cytotoxic effects of chemotherapy agents, such as 5-fluorouracil [116].

A recent study explored the expression of the stem cell-associated intermediate filament nestin in primary EC specimens and showed that positive tumors had significantly shorter median survival than those with nestin-negative tumors; furthermore, nestin phenotype correlated with the expression of proliferation-associated markers in tumor specimens [117].

Esophageal cancer stem cells could be involved in the mechanism of tumor metastasis. In line with this hypothesis, Chen and coworkers observed higher levels of Placental Growth Factor (PLGF) and Metalloproteinase (MMP9) in metastatic than in non-metastatic esophageal cancer [118]. Using an esophageal cancer cell line, te-1, it was possible to show that PLGF stimulates MMP9 expression in esophageal cancer cells and PLGF-positive esophageal cancer cells grow when transplanted into recipient mice, where they grow much faster than PLGF-negative cells [118]. According to these findings it was suggested that CSCs in esophageal cancer may release PLGF to promote cancer metastasis through MMP9 activation [118].

Some studies have investigated the sensitivity of esophageal cancer stem cells to radiation therapy. In this context, in an initial study Zhang and coworkers have shown that some radiation-resistant tumor cells exhibited characteristics of cancer stem cells after fractionated irradiation of esophageal cancer cells [119]. The radioresistant cells displayed several stem-like properties, such as increased telomerase activity, enrichment of side-population cells, expression of the stem cell markers beta-catenin, 3–4 October and beta (1) integrin [119]. In another study, Wang and coworkers have shown that tumorspheres isolated from esophageal cancer cell lines are more radioresistant than the parental lines from which they were derived [120]. Following exposure of tumor spheres to radiations an increase of stem-like CD271^+^/CD44^+^ cells was observed [120].

As above mentioned, NOTCH signaling is frequently activated in EACs. A recent study provided evidence that NOTCH activity is required for stemness and tumorigenicity of EAC. Several lines of evidence support this view: NOTCH signaling is greater in less differentiated EACs than in differentiated EACs; NOTCH activity is required for tumor growth in esophageal adenocarcinoma xenograft models; the growth and the expression of CSC markers is inhibited in esophageal adenocarcinoma cell spheres; esophageal adenocarcinoma CSC population is more sensitive to inhibition of NOTCH than bulk tumor cells [121].

The study of animal models of esophageal cancer helped also to identify and characterize cancer stem cells. Thus, studies aiming to define the oncogenic role of Acylglycerol Kinase (AGK) in squamous epithelium, provided evidence that AGK overexpression induced constitutive JAK2/STAT3 activation, promoting the expansion of a population of cells with stem cell features, including CD44 expression, SP and expression of pluripotency-associated markers such as SOX2, OCT4, NANOG, BMI1, ABCG2 [122]. Another study explored a model of squamous epithelium tumorigenesis based on the study of the cooperation between SOX2 and microenvironment signals [123]. SOX2 overexpression induces the expansion of basal progenitors with development of a premalignant lesion that progresses to carcinoma in the presence of an inflammatory stimulus that induces Stat3 activation [123]. In addition to SOX2, also the expression of SOX9 seems to be essential for the induction and maintenance of stem properties of esophageal CSCs. In fact, it was provided evidence that the YAP1 transcription co-activator directly up-regulates SOX9 and endows CSC properties in a wide variety of non-transformed cells of gastrointestinal origin, including primary esophageal epithelium cells [124]. In these cells, CSC properties, including tumorsphere formation, propagation and tumorigenicity are YAP1 and SOX9-dependent [124]. YAP1 upregulates also EGFR expression is esophageal cancer cells, acting at the transcription level through binding to the EGFR promoter. YAP1 overexpression stimulates cell proliferation and confers resistance to therapy. Importantly, YAP1 inhibitors sensitize esophageal cancer cells to cytotoxic agents and may offer a strategy to bypass chemoresistance in these cells [125]. A link between EGFR and esophageal CSCs is supported also by other studies. ESCC cancer stem cells express high levels of CD44; cancer cells with high CD44 expression show typical features of EMT. EGFR plays a key role in EMT induction in CD44^+^esophageal cancer cells though TGF-β [126]. Epithelial-mesenchymal transition is of critical importance for the generation of cancer stem cells and EGFR inhibitors block EMT at the invasive front of ESCCs [126].

The Wnt/β-catenin pathway is an important regulator of esophageal CSCs. The aberrant β-catenin expression and consequent Wnt pathway activation are determinants of ESCC cancer progression, invasion and metastasis. Two recent studies support a role for Wnt/β-catenin pathway activation in the maintenance of CSCs in ESCC. Thus, in a first study it was reported that miR-942 expression promotes ESCC stem cell traits by activating the Wnt/β-catenin pathway through a targeting effect on the inhibitors of this pathway (glycogen synthase kinase 3 beta, transducing-like enhancer protein 1 and frizzled-related protein 4) [127]. miR-942 was overexpressed in ESCC and its level of expression negatively correlated with prognosis [128]. In a second study, it was provided evidence that the Wnt Ligand WNTA promotes an invasive and self-renewing phenotype in ERSCCs and stimulates the generation of CD44^high^ALDH1A^+^ cells exhibiting cancer stem-like properties [129]. In support of this observation it was also observed that WNT6 expression in ESCC primary tumors is a predictive factor of negative outcome [127].

In parallel with the study and characterization of EC stem like cells, the development of clinically relevant xenograft models is of fundamental importance. However, very few studies have reported the successful xenotransplantation of primary tumor cells issued either from EAC or ESCC. Initial studies have reported the sporadic successful establishment of primary xenografts injecting primary tumor cells derived from EAC or ESCC into nude mice [130,131,132]. These studies have analyzed a limited number of primary tumors and do not have identified factors that could affect tumor engraftment in immunodeficient mice. Two recent studies have analyzed the engraftment of primary EC into NOD/SCID mice analyzing larger numbers of primary tumors and attempting an analysis of the factors that could affect tumor engraftment into immunocompromised mice. Thus, Dodbiba and coworkers have analyzed the engraftment of 70 primary ECs into NOD/SCID mice showing that 32% of these cases show engraftment; the rate of engraftment was highly comparable for both EACs and ESCCs [133]. The analysis of the tumor-related factors potentially influencing the rate of engraftment showed that for EACs preferentially engrafted poorly differentiate tumors and for both EACs and ESCCs preferentially engrafted tumors of patients not performing adjuvant therapy before surgical debulking [133]. The pathological analysis of xenografts showed that tissue morphology remained similar between passages and usually correlated well with the original patient tumor [133]. These findings were confirmed in a second study by the same authors, reporting the successful xenografting of 22/55 implanted esophageal cancer specimens: engraftment was associated with poorly differentiated tumors and older patients [134]. The implanted tumors represent stable and useful models for drug testing, with all the limitations of the xenotransplantation assays related to patient selection due to limited rate of engraftment, limitations in the reproduction of tumor heterogeneity and inappropriate reproduction of the tumor environment [134]. A second recent study was carried out on 96 primary ESCC samples derived from Chinese patients providing evidence about a rate of engraftment corresponding to 38.5% with similar rates of engraftment in patients whose tumors display or not PI3KCA mutations [135]. The presence of HER2 overexpression, due to gene amplification or not, was associated with absent engraftment in xenotransplantation assay [135].

## 6. Conclusions

The progress in molecular characterization of esophageal cancer provided fundamental data supporting that the histological subtypes EAC and ESCC are two distinct entities for all the molecular features analyzed. Although many of the same pathways are somatically altered in EACs and ESCCs, the specific genes affected are different, indicating distinct physiopathologic mechanisms and suggesting the need for different therapeutic approaches. For their molecular features, EACs mostly resemble the chromosomal instability subtype of gastric cancer, while ESCCs resemble other squamous carcinomas. These observations indicate that in studies of neoadjuvant, adjuvant or systemic therapies, EACs and ESCCs must be considered separate entities and cannot be combined. According to these molecular findings, mouse models suggest that Barrett’s esophagus and EAC may originate from proximal gastric cells or embryonic remnant cells located at the gastro-esophageal junction.

On the other hand, the molecular studies indicate also that the genetic alterations observed in esophageal cancers are multiple and complex and a targeting of a single druggable genetic abnormality in this tumor is not sufficient to achieve the disease control. An additional problem is related to intra-tumor heterogeneity of both EACs and ESCCs, which fosters tumor evolution, and is a key challenge in response to drug treatment.

The cellular studies have suggested the existence of a rare population of immature cancer cells that initiate and maintain the neoplastic process. These cells display a high metastatic potential and resistance to conventional anti-cancer therapy.

The actual standard of care for patients with stages II-III EC involves chemotherapy, or chemoradiation either in the neoadjuvant or adjuvant setting of surgical resections of esophagus. Targeted therapies (anti-receptor tyrosine-protein kinase Erb-2 (HER2) or anti-Epidermal growth factor receptor (EGFR)) are sometimes used in concert with chemoradiation. However, the success of this therapeutic approach is limited in that the average 5-year survival of EC is less than 18%. In this context, given the progress in the understanding of the molecular abnormalities of EACs and ESCCs, it is expected the rapid development of a novel arsenal of therapeutic options in esophageal cancers. In parallel, oncologists, surgeons, pathologists and scientists can contribute to optimize the first-line therapy of ECs to try to improve survival rates. A straight-forward solution to this problem would imply a utilization in clinical studies of molecular classification and diagnostics of ECs, with identification of new therapeutic targets.

## Figures and Tables

**Table 1:** Membrane markers of esophageal cancer stem cells.

Marker	Function	Biological properties	Assays	Subpopulations	Clinical Significance
ABCG2 (ATP-Binding Cassette sub-family G member 2)	This membrane protein functions as a xenobiotic transporter and may have a role in multi drug resistance.	Tumor spheres isolated fromEC cell lines are enriched in ABCG2^+^ cells. Cigarette smoke condensate induces ABCG2 expression and potentiates CSC properties.	ABCG2^+^ cells isolated from EC cell lines and EC biopsies form tumor-spheres in vitro and tumors in vivo in nude mice.	ABCG2^+^/ALDH1^+^ cells are tumorigenic.	ABCG2 is overexpressed in ESCC, in association with tumor grade, stage and metastasis. 5-FU treatment increases ABCG2^+^ cells; 5-FU+COX2 inhibitor decreases ABCG2^+^ cells.
CD44 Also, known as HCAM (Homing Cell Adhesion Molecule)	CD44 is a cell surface glycoprotein involved in cell-cell interactions, cell adhesion and migration.	CD44^+^/CD24^-^ cells isolated from EC cell lines and EC biopsies have phenotypic and functional properties of CSCs.	CD44^+/^CD24^-^ cells isolated from EC cell lines and EC biopsies form tumor-spheres in vitro and tumors in vivo in nude mice.	CD44^+^/CD24^-^ cells have high sphere-forming potential, are resistant to irradiation, reside in hypoxic niches, formed tumors in xenotransplantation assays and their number correlated with tumor grade. CD44^+^/ICAM1^+^, CD44^+^/ALDH1^+^ and CD44^+^CD133^+^ have high CSC potential.	The number of CD44^+^/CD24^-^ cells correlated with response of residual EAC to chemo/radiation. CD44 expression increases during the progression of Barrett’s metaplasia to EAC.
CD90 Also, known as Thy-1	CD90 is a heavily glycosylated, glyco- Psphatidylinositol anchored cell surface protein. CD90 is used as a marker for a variety of stem cell populations.	CD90^+^ cells isolated from cancer cell lines have CSC properties. CD90 expression is increased in esophageal tumor tissues, compared to normal ones.	CD90^+^ cells isolated from KYSE140 or KYSE520 cell lines form tumor-spheres in vitro and tumors in vivo in nude mice.	Only a minority of CD90^+^ cells co-express CD271 or CD44.	CD90 expression is upregulated in primary ESCC cells. 2–10% CD90^+^ cells in ESCCs. CD90^+^ cells are radio-resistant.
CD133 Also, known as Promin-1	CD133 is a membrane glycoprotein, encoded by the PROM1 gene, member of the pentasman transmembrane glycoproteins.	CD133^+^ or CD133^+^/CXCR4^+^ cells isolated from EC cell lines and EC biopsies have phenotypic and functional properties of CSCs.	CD133^+^/CXCR4^+^ cells isolated from TE-1 cell line have a high colony-forming capacity.	CD133^+^/CXCR4^+^ cells have CSC properties with high proliferative potential.	CD133 expression associated with lymph node metastasis, clinical stage and histo- pathological grade. 21% of ESCCs have high CD133^+^/CXCR4 expression, associated with negative prognosis. CD133 expression increases during the progression of Barrett’s metaplasia to EAC.
CD271 Also, known as p75 Neutrophin Receptor (p75NTR)	CD271 is the low-affinity nerve growth factor receptor, one of the two receptor types for neutrophins.	CD271^+^ cells isolated from cancer cell lines (KYSE70) have CSC properties.	CD271^+^ cells isolated from EC cell lines and EC biopsies form tumor-spheres in vitro and tumors in vivo in nude mice.	CD271^+^/CD90^+^, CD271^+^/CD44^+^ cells have the same CSC function, compared to CD271^+^/CD90^-^ , CD271^+^/CD44^-^ cells. Quiescent CD271^+^ cells express ABCG2 and are chemoresistant.	CD271 expression is higher in ESCC tissue than in normal esophageal epithelium. High CD271 expression is observed in 34% of ESCCs.
ITGA7 (Integrin alpha 7)	ITGA7 is a member of the integrin family of cell surface proteins that mediate cellular interactions with the extracellular matrix. Together with a beta subunit, it forms the functional receptor that binds laminin.	ITGA7^+^ cells isolated from cancer cell lines have CSC properties. ITGA7 overexpression pro- Motes CSC properties, metastasis and EMT. ITGA7 knockdown decreases CSC properties.	in vitro: tumor-sphere In vivo: xenotranspanta- tion in nude mice.	ITGA7 and CD90 are co-expressed. ITGA7^+^/CD90+ cells have a high clono- genic potential.	ITGA7 expression is associated with poor prognosis in ESCC, poor differentiation and lymph node metastasis. Chemotherapy enriches ITGA7^+^ cells.
